# Basic auditory processing and sensitivity to prosodic structure in children with specific language impairments: a new look at a perceptual hypothesis

**DOI:** 10.3389/fpsyg.2015.00972

**Published:** 2015-07-10

**Authors:** Ruth Cumming, Angela Wilson, Usha Goswami

**Affiliations:** Department of Psychology, Centre for Neuroscience in Education, University of CambridgeCambridge, UK

**Keywords:** SLI, phonology, auditory processing, rise time, oscillations

## Abstract

Children with specific language impairments (SLIs) show impaired perception and production of spoken language, and can also present with motor, auditory, and phonological difficulties. Recent auditory studies have shown impaired sensitivity to amplitude rise time (ART) in children with SLIs, along with non-speech rhythmic timing difficulties. Linguistically, these perceptual impairments should affect sensitivity to speech prosody and syllable stress. Here we used two tasks requiring sensitivity to prosodic structure, the DeeDee task and a stress misperception task, to investigate this hypothesis. We also measured auditory processing of ART, rising pitch and sound duration, in both speech (“ba”) and non-speech (tone) stimuli. Participants were 45 children with SLI aged on average 9 years and 50 age-matched controls. We report data for all the SLI children (*N* = 45, IQ varying), as well as for two independent SLI subgroupings with intact IQ. One subgroup, “Pure SLI,” had intact phonology and reading (*N* = 16), the other, “SLI PPR” (*N* = 15), had impaired phonology and reading. Problems with syllable stress and prosodic structure were found for all the group comparisons. Both sub-groups with intact IQ showed reduced sensitivity to ART in speech stimuli, but the PPR subgroup also showed reduced sensitivity to sound duration in speech stimuli. Individual differences in processing syllable stress were associated with auditory processing. These data support a new hypothesis, the “prosodic phrasing” hypothesis, which proposes that grammatical difficulties in SLI may reflect perceptual difficulties with global prosodic structure related to auditory impairments in processing amplitude rise time and duration.

## Introduction

Specific language impairment (SLI) is a neurodevelopmental disorder of learning that affects the processing and production of spoken language (Leonard, 2014). Children with SLI have no obvious hearing or neurological impairments, and no apparent prosocial difficulties, yet they fail to acquire language skills at an age-appropriate rate. A hallmark of SLI is grammatical difficulties, usually described as difficulties with morpho-syntax (see Leonard, 2014, for a recent overview). For example, children with SLI will fail to use inflectional endings appropriately (“She comb her hair”), they will fail to mark tense (“Yesterday I fall down”), and they show poor understanding of syntactic devices like word order, selecting a picture of a fish eating a man for the sentence “The fish is eaten by the man” (see Hsu and Bishop, 2011). Rice and colleagues established that a composite measure of awareness of tense-related morphemes was able to distinguish 5-year-old English-speaking children with SLIs with a sensitivity of 97% and a specificity of 98% (Rice, 1998).

It has proved surprisingly difficult to establish a sensory/perceptual basis for SLI, and accordingly currently-prominent theories are in the linguistic/grammatical domain. For example, it is proposed that linguistic principles such as tense marking may be slow to mature in SLI (Rice and Wexler, 1996), or that there may be an inherited grammatical deficit linked to a genetic impairment in processing “extended” grammatical representations (those that are nonlocal, hierarchical, abstract, and generated by the child, rather than the “local” grammatical representations thought to be copied from current discourse; see van der Lely and Pinker, 2014). Other theories propose that children with SLIs may have deficits in statistical or procedural learning, which compromise the extraction of implicit grammatical rules (e.g., Ullman and Pierpont, 2005), or that the primary impairment lies with knowledge of implicit rules for marking tense, number, and person (Gopnik and Crago, 1991).

A systematic programme of cross-language research exploring morpho-syntactic, procedural-deficit or exemplar-learning theories has yet to emerge. Nevertheless, where available, studies that compare children with SLIs who are learning different languages do not support strong grammatical theories. For example, theories arising from English data that specify a particular difficulty with tense marking or agreement have found mixed support in studies of children with SLIs in other European languages (e.g., Kunnari et al., 2011, Finnish; Leonard et al., 2012, Hungarian). Indeed, after a careful comparison of children with SLIs learning English, Italian, Hebrew, Spanish, and Swedish, Leonard (2000) noted that most grammatical errors were language-specific, and that children with SLI who were acquiring languages with richer inflectional morphologies seemed to make fewer errors. This lack of cross-language universality in the aspects of morpho-syntax that are affected in SLI, along with new insights into how the brain encodes language, suggest that it is worth looking again at potential sensory/perceptual causes of developmental SLIs.

**Figure 1:**
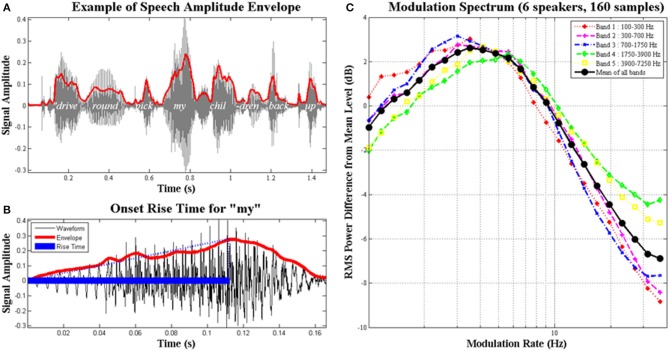
**The amplitude envelope of speech, syllable rise times and the modulation spectrum**. Schematic depiction of **(A)** the amplitude envelope (AE) for the phrase “..drive round, pick my children back up..,” the AE is in red and the original signal is in gray; **(B)** the rise time for the syllable “my,” shown as blue filled and dotted lines; the *rise time* is the time taken for the envelope to reach its highest amplitude; and **(C)** averaged long-term modulation spectra of 160 conversational speech samples from six different speakers; speech samples were between 24 and 34 s in length. **(C)** shows the modulation spectra for five different frequency bands in speech with the average in black; the AE in adult speech is clearly dominated by amplitude modulations at the temporal rate of syllable production (3–5 Hz), hence the most prominent energy changes convey information about syllable pattern. Figure reproduced from Goswami and Leong (2013).

In developmental dyslexia, a childhood disorder of learning that has received intensive cross-language scrutiny (Ziegler and Goswami, 2005), the investigation of auditory sensitivity to amplitude envelope rise time has proven theoretically fruitful across languages (Goswami, 2011, 2015; see Figure 1). The amplitude envelope (AE) contains patterns of amplitude modulation (AM) at different temporal rates, and carries information about speech rhythm and prosodic structure. The AE of adult-directed speech is dominated by modulations at 4–6 Hz, the “syllable” rate, as most adult speakers produce around five syllables per second (shown in Figure 1, see also Ghitza and Greenberg, 2009). The AE of child-directed speech is dominated by modulations at both ~2 Hz and ~5 Hz (Leong and Goswami, 2014; designated the “stressed syllable” and “syllable” rates). Dauer (1983) showed that 2 Hz is the approximate rate of stressed syllable production across languages (see also Ghitza and Greenberg, 2009). In rhythmic child-directed speech, for example nursery rhymes, Leong et al. (2014) showed that syllable prominence is determined by *phase alignment* of these two AM rates. Thus the perception of prosodic prominence and English speech rhythm depends on the modulation peaks at the “stress” and “syllable” AM rates being aligned in the spoken signal. As noted by Goswami and Leong (2013), accurate AM rise time perception would appear critical for successful AM phase alignment and the accurate perception of speech rhythm.

Meanwhile, neural studies of speech encoding by adults show that AM rise times are important for re-setting the cortical oscillations that are thought to encode the speech signal in auditory cortex (e.g., Gross et al., 2013; Doelling et al., 2014). Endogenous cortical oscillations at different temporal rates re-set their activity using ARTs to be “in phase” with the AMs in the speech signal at corresponding temporal rates, thereby aligning neuronal oscillatory peaks with AM peaks in the signal. This process of “phase alignment” enables successful neural entrainment to the signal and contributes to speech intelligibility (Giraud and Poeppel, 2012, for review). Adult studies show that the phase alignment (or “phase-locking”) of brain rhythms and speech rhythms plays a critical role in language comprehension (e.g., Luo and Poeppel, 2007; Luo et al., 2010; Peelle et al., 2010). As sensitivity to amplitude envelope rise time (hereafter ART) is important for the successful neural encoding of language, reports of impaired sensitivity to ART in children with SLIs deserve further investigation (Corriveau et al., 2007; Fraser et al., 2010; Beattie and Manis, 2012; Goswami, 2015).

The first study to examine sensitivity to ART in children with SLI was conducted by Corriveau et al. (2007). They examined auditory processing of non-speech tones with different ARTs, sound intensity and sound duration in 21 10-year-olds with SLI and in 21 chronological-age (CA) matched and 21 younger language-age (LA) matched controls. The children with SLI exhibited marked impairments in auditory processing of ART and sound duration, but not sound intensity. The ART and duration measures also accounted for significant unique variance in measures of vocabulary and phonological awareness when age, NVIQ and attention were controlled statistically. Corriveau et al. proposed that auditory insensitivity to cues important for the perception of rhythm and stress might impair accurate prosodic processing in SLI, with important consequences for language development. Fraser et al. (2010) used the same non-speech tone measures of ART and sound intensity in a study comparing 10-year-old children with SLI and intact reading skills to matched children with SLI and impaired reading skills and typically-developing (TD) children. Children with developmental dyslexia (intact language, impaired reading) were also studied. Fraser et al. reported that sensitivity to ART was impaired in the two groups with impaired phonology, but not in the “pure” SLI group (although ART thresholds were elevated in this group). Auditory processing of simple intensity was preserved in all groups. In regression analyses, ART showed unique associations with phonological development in the sample, but not with “non-phonological” language measures (i.e., vocabulary, grammatical morphology and sentence processing). Although these latter findings differ from those reported by Corriveau et al. (2007), Fraser et al.'s sample of children showed significantly less impaired language skills compared to Corriveau et al.'s sample [the averaged scaled Clinical Evaluation of Language Fundamentals (CELF) scores were 5.24 and 3.74, respectively, *p* < 0.001]. Further, Fraser et al. reported that even the children with SLI who had intact reading skills in their sample showed significant phonological difficulties. However, prosodic phonology was not measured in this study.

Beattie and Manis (2012) studied children with both reading and language impairments and children with reading impairments only, and used the same non-speech tone measures of ART and intensity discrimination as Corriveau, Fraser and colleagues. They also reported that both groups of children were impaired in perceiving ART compared to CA controls. Finally, Richards and Goswami (2015) gave measures of auditory sensitivity to non-speech tones varying ART, sound duration, sound intensity and sound frequency to a small sample of 12 children with pure SLI and 10 CA controls. They also gave the children two measures of prosodic sensitivity, the DeeDee task (Whalley and Hansen, 2006) and a Lexical Stress task based on imageable multi-syllabic words (e.g., ladybird, umbrella). In the DeeDee task, children heard the names of different cartoon and book characters (shown in pictures) as a series of stressed (DEE) or unstressed (dee) syllables, for example the name “Harry Potter” (strong-weak-strong-weak, SWSW) would be DEEdee DEEdee. Children heard two DeeDee sequences for each picture, and were asked to choose the prosodic match. In the Lexical Stress task, children saw a picture (e.g., ladybird), and heard two tokens of the picture name, one stressed correctly (‘ladybird) and one stressed incorrectly (lady'bird). The child was asked to indicate the token pronounced correctly. Richards and Goswami found that the children with SLI were impaired in both prosodic tasks. The children with SLI also showed impaired sensitivity to both ART and duration, although not to simple sound frequency or intensity. In regression analyses, ART was the only significant predictor of performance in the Lexical Stress task by children with SLI, although sensitivity to sound frequency approached significance as a predictor (*p* = 0.075). In this relatively small sample, none of the auditory variables were significant predictors of DeeDee performance.

These four studies suggest that the perception of ART is impaired in children with SLI. Furthermore, the study by Richards and Goswami (2015) provides some evidence that this auditory impairment is related to performance with prosodic phonology. The same relationship between ART and prosodic sensitivity has been found in studies of children with developmental dyslexia (Goswami et al., 2010, 2013). The findings for rise time sensitivity and prosodic phonology also complement the relationships demonstrated between rise time sensitivity and non-prosodic phonology (syllable, rhyme, and phoneme awareness) in the earlier SLI studies (Corriveau et al., 2007; Fraser et al., 2010). Impaired access to prosodic phonology in SLI would be expected to adversely affect language development (see Corriveau et al., 2007) and to affect levels of phonological awareness that are “downstream” to prosody in the phonological hierarchy (see Goswami, 2015). Further, auditory perceptual impairments that reduce the child's access to prosodic structure could be one cause of the well-documented impairments in morpho-syntax in English-speaking children with SLI. Morphological information such as grammatical inflection and tense agreement is usually carried by unstressed syllables. Indeed, a prosodic theory of SLI was suggested 20 years ago by Leonard and his colleagues, when investigating similarities between English and Italian children with SLI (e.g., McGregor and Leonard, 1994; Bortolini and Leonard, 1996).

In essence, the proposal made by Leonard and colleagues (the “surface” hypothesis) was that patterns of strong and weak syllables characterize all languages, and that children with SLI might have difficulties in perceiving less prominent syllables because of perceptual difficulties with sounds of short duration and low intensity. These less-prominent syllables are often function words or syllables that carry grammatical morphemes. Leonard and colleagues pointed out that English-learning infants extract the strong-weak (SW) prosodic template characterizing many English nouns (“baby,” “bottle”) by around 7 months (Jusczyk et al., 1999) and use this cue in speech segmentation. The SW template also plays an important role for English-speaking children in early language production, where unstressed rather than stressed syllables are typically omitted (e.g., “banana,” WSW, is typically “nana,” SW). Accordingly, Leonard and colleagues suggested that children with SLI may find it difficult to perceive weak syllables when they do not follow strong syllables, as in these circumstances they cannot organize the weak syllable using the proto-typical SW English template. Hence “the” is more likely to be omitted in a sentence like “The car is red” (WSWS) than “Jill pushed the car” (SSWS), where it follows the strong syllable “pushed.”

Despite generating cross-language evidence in support of this version of a perceptual theory (e.g., McGregor and Leonard, 1994; Leonard and Bortolini, 1998), Leonard subsequently decided that the theory could not account for all the evidence (e.g., Leonard and Dispaldro, 2013). For example, Italian children with SLI also make errors with morphemes that should be salient according to the theory (e.g., reducing'pecora (sheep) to ‘peca; for this SWW item, they should omit the final W syllable, and say ‘peco). However, in Leonard's writings acoustic prominence was considered largely in terms of syllable duration and overall amplitude. As outlined above, new perspectives on speech rhythm (Leong et al., 2014) and neural speech encoding (Gross et al., 2013; Doelling et al., 2014) suggest that the detection of syllable prominence depends on sensitivity to ARTs and on the successful phase alignment of neural networks to AMs in speech at delta (~ 2 Hz) and theta (~ 5 Hz) rates. Hence the acoustic cues of duration, frequency and simple intensity may be less important in detecting prosodic prominence than traditionally supposed (see also Greenberg et al., 2003; Kochanski et al., 2005, for experimental data). Further, ART is greater for stressed syllables. As shown by Leong et al. (2014), accurate perception of prominent syllables, as well as accurate perception of the prosodic patterns of strong and weak syllables governing speech rhythm, depends in part on the temporal alignment of the modulation peaks at two AM rates dominant in child-directed speech, the “stress” rate of ~2 Hz, and the “syllable” rate of ~5 Hz. Hence perceptual sensitivity to changes in ART at these relatively slow (in speech processing terms) temporal rates might be critical if children are successfully to detect prosodic prominence.

Accordingly, a new form of the prosodic hypothesis informed by neural data may provide a useful perceptual approach to understanding SLIs across languages. This form of prosodic hypothesis, termed here the “prosodic phrasing” hypothesis, would apply to global prosodic structure and speech rhythm patterns rather than SW templates *per se* (see Frazier et al., 2006). Frazier et al. (2006) argued that words in language are grouped together into phrases by their rhythmic and durational properties and by their tonal pitch, and that this global patterning of prosodic phrasing was critical to language comprehension. Every language uses prosodic grouping and prosodic prominence, albeit in different ways, and so children's perception of global patterning would relate to their comprehension in every language. Accordingly, a perceptual difficulty with ART (by hypothesis, present from birth) would impair perception of this global patterning, and would thereby impair morphological development. According to the prosodic phrasing hypothesis, children with SLIs would have difficulties in extracting grammatical morphology when speech rhythm patterns failed to support perception of the weaker syllables that carry morphological information.

As noted by Frazier et al. (2006), when syllables are organized prosodically, they become part of a larger lexical structure or utterance of precedence and prominence relations. The same utterance can be divided into semantic units using one set of rules, into syntactic units using another set of rules, and into phonological units using another set of rules—termed the linguistic “binding problem.” Frazier et al. argued that if each level of linguistic representation was indexed to the prosodic representation, then a linguistic unit such as “past tense” could be identified across *different* representations. Accordingly, the prosodic representation is the skeletal structure upon which understanding the utterance depends. This notion is similar to the developmental proposal made by Goswami and Leong (2013) concerning the key role of quasi-periodic “skeletons” of syllable beats in language acquisition. Goswami and Leong argued that neural entrainment to syllable beats was one important foundation for language acquisition, and that efficient sensory processing of the temporal positions of the syllable “beats” in speech was related to ART sensitivity. Successful beat processing was thought to enable the successful extraction of prosodic structure during language acquisition, and was argued to be compromised in developmental dyslexia (Goswami and Leong, 2013).

Here we extend this notion to developmental SLIs, via the “prosodic phrasing” hypothesis. We propose that impairments in processing ART in children with SLIs compromises the extraction of the higher-order global prosodic patterns in utterances, which leads to sensory-based language-specific patterns of errors with morpho-syntax during language acquisition. At the level of neural entrainment, the perceptual difficulties with ARTs experienced by children with SLI would be expected to affect successful entrainment to the rhythm patterns in speech. Goswami and Leong (2013) noted that universal neural oscillatory processes track temporal regularities and encode the beat distribution patterns in speech, utilizing perceptual cues such as stressed syllables and P-centers (see also Kotz and Schwartze, 2010). In the prosodic phrasing hypothesis, these perceptual difficulties apply to the prosodic and rhythmic pattern of the global utterance rather than specifically to the SW prosodic template. Hence in developmental SLIs, impaired perception of prosodic phrasing would impair utterance comprehension and lead to morpho-syntactic errors during language production. Rather than affecting specific grammatical morphemes across all utterances, a prosodic difficulty related to the auditory processing of ART and the perception of prosodic phrasing should cause systematic errors depending on the global prosodic patterning of specific individual utterances.

One clear test of this prosodic phrasing hypothesis is that the perception of lexical and metrical stress as well as ART should be impaired in children with SLIs. To our knowledge, this has not yet been studied directly in relation to ART, apart from in the study by Richards and Goswami (2015). While difficulties with stress have been studied by some developmental researchers, the conclusion is often that prosodic difficulties do not represent a core impairment in SLI (e.g., Marshall et al., 2009). By contrast, sensitivity to metrical stress patterns and rhythm patterns have been investigated intensively in children with developmental dyslexia, where prosodic processing does seem to represent a core impairment (see Goswami, 2015, for a recent summary). As well as showing prosodic and rhythmic deficits, children with dyslexia also show difficulties in perceiving ART and exhibit associated neural difficulties in entrainment to rhythmic language (Power et al., 2013).

To see whether similar behavioral profiles would characterize children with SLI, here we utilized two prosodic measures from our dyslexia studies, a measure of direct stress perception (recognition of the mis-stressing of four-syllable words, see Leong et al., 2011; Goswami et al., 2013) and the DeeDee task (see Goswami et al., 2010, 2013). We also measured auditory sensitivity to ART, sound duration and rising f0, using both non-speech (tone) and speech (synthetic “ba” syllable) stimuli. Measuring auditory sensitivity to these acoustic cues in speech stimuli is novel, and should enable a more comprehensive understanding of whether an auditory/perceptual theory of SLIs based on ART and prosodic processing has merit. Note that a successful perceptual approach to SLIs should apply across languages.

## Methods

### Participants

Ninety-five children aged on average 9 years 6 months participated in this study. Due to the large number of measures, testing took place over two consecutive academic years (hereafter, Year 1 and Year 2 testing). Forty-five of the children were referred by their schools as having a specific language impairment. Only children who had no additional learning difficulties (e.g., dyspraxia, ADHD, autistic spectrum disorder, dyslexia) and English as the first language spoken at home were included. The absence of additional learning difficulties was based on the reports of teachers and speech and language therapists in schools, and our own testing impressions of the children. Ethical approval for the study was obtained from the University of Cambridge Psychology Research Ethics Committee.

All children received a short hearing screen using an audiometer. Sounds were presented in both the left and the right ear at a range of frequencies (250, 500, 1000, 2000, 4000, 8000 Hz), and all children were sensitive to sounds within the 20 dB HL range. Forty-five of the children (31 male, 14 female; mean age 9 years, 6 months) either had a statement of SLI from their local education authority, or had received special help for language via the teacher(s) with responsibility for special educational needs in school, and/or showed severe language deficits according to our own test battery. These children (SLI group) were drawn from a number of schools via language support units in the schools, referral to the study by speech and language therapists or referral by teachers with responsibility for special educational needs. All SLI children were assessed experimentally in Year 1 using two expressive and two receptive subtests of the Clinical Evaluation of Language Fundamentals-3 (CELF-3; Semel et al., 1995), and were included in the study if they scored at least 1 *SD* below the mean on two or more of these subtests. Individual standardized scores of the children in the SLI group for the four CELF-3 subtests administered, as well as receptive vocabulary as measured by the British Picture Vocabulary Scales (BPVS, Dunn et al., 1982), and nonverbal IQ as measured by the Wechsler Intelligence Scales for Children (WISC-III; Wechsler, 1992) or Raven's Standard Progressive Matrices (Plus version, Raven, 2008), all measured in Year 1, are shown in Table 1. The table also shows single-word reading and spelling scores on the British Ability Scales (BAS; Elliott et al., 1996) and the Test of Word Reading Efficiency (TOWRE; Torgesen et al., 1999), which were administered in Year 2.

**Table 1:** **Full SLI sample showing pure SLI and SLI PPR sub-groups**.

**SLI sub-group**	**BPVS^a^**	**CELF expressive^b^**		**CELF receptive**		**NIVQ^c^**	**Reading and spelling**		
		**FS**	**SA/WS**	**CD**	**SR/SS**		**TOWRE^d^**	**BAS read^e^**	**BAS spell^e^**
Pure	107	8	5	9	6	131	101	114	118
Pure	78	5	6	12	7	125	111	117	127
Pure	96	11	5	12	6	119	118	106	99
Pure	85	4	4	3	4	110^f^	89	105	87
Pure	93	7	7	7	7	110^f^	101	99	109
Pure	80	5	7	6	4	105^f^	95	86	89
Pure	92	5	6	4	10	105^f^	87	78	91
Pure	98	6	5	11	10	103	90	108	105
Pure	104	11	6	6	10	100	101	112	106
Pure	106	5	5	4	3	97	114	118	118
Pure	84	4	4	5	5	95^f^	103	106	131
Pure	89	3	6	4	5	90^f^	96	103	90
Pure	90	7	5	3	11	90^f^	N/A	86	93
Pure	104	9	4	7	5	88	119	110	105
Pure	100	8	3	6	4	81	93	94	90
Pure	101	3	4	6	5	80^f^	93	86	61
PPR	77	3	4	8	7	115^f^	68	74	66
PPR	101	3	6	6	11	112	69	82	68
PPR	86	3	3	6	4	105^f^	80	79	91
PPR	81	3	4	3	5	100	60	55	55
PPR	72	2	4	4	5	97	64	69	60
PPR	90	6	4	8	13	97	80	82	78
PPR	74	3	5	5	5	95^f^	79	85	77
PPR	78	3	3	3	5	95^f^	82	81	79
PPR	89	2	4	6	12	94	87	81	78
PPR	97	5	3	3	3	94	N/A	55	55
PPR	107	3	7	6	6	94	72	80	78
PPR	76	2	3	6	4	91	63	69	59
PPR	90	3	3	3	4	85	56	64	63
PPR	91	5	3	6	6	81	65	74	63
PPR	90	3	3	6	6	80^f^	71	79	76
	87	1	6	1	2	75	N/A	55	59
	100	8	4	8	6	75^f^	101^g^	96	89
	85	3	3	6	6	75^f^	74	77	79
	89	1	1	1	6	75^f^	54	64	69
	83	8	3	3	4	70^f^	94^g^	92	101
	80	3	3	3	4	75^f^	102^g^	96	97
	76	3	3	4	3	70^f^	54	67	65
	73	3	4	3	4	70^f^	54	56	62
	87	3	2	4	4	65^f^	62	70	74
	86	5	4	10	12	65^f^	68	71	75
	72	3	3	3	3	60^f^	N/A	55	55
	90	3	3	3	3	57	N/A	55	55
	59	3	3	3	3	55^f^	N/A	55	55
	64	3	3	6	5	55^f^	84	82	90
Mean (*SD*)	87.49 (11.38)	4.44 (2.44)	4.13 (1.39)	5.38 (2.62)	5.84 (2.78)	96.05 (49.56)	83.44 (33.58)	82.48 (22.40)	82.00 (20.65)

*N/A means score not available, either as the child was absent or as the child refused to try the non-word component of the TOWRE, which is a timed test*.

a *British Picture Vocabulary Standard Score (M = 100, SD = 15)*.

b *Clinical Evaluation of Language Fundamentals (CELF) Expressive and Receptive Sub-tests (M = 10, SD = 3); FS, Formulating Sentences; SA, Sentence Assembly; WS, Word Structure; CD, Concepts and Directions; SR, Semantic Relations; SS, Sentence Structure*.

c *Higher Standard Score Non-Verbal IQ from WISC or Ravens (M = 100, SD = 15)*.

d *Test of Word Reading Efficiency combined Standard Score (M = 100, SD = 15)*.

e *British Ability Scales Standard Score (M = 100, SD = 15)*.

f *Ravens SS shown instead of WISC SS*.

g *Low NVIQ children with preserved reading skills. These three participants showed average phonological skills (mean oddity score = 9/20; mean PSTM score = 27/64)*.

Note that in our prior studies of ART and sensitivity to syllable stress in dyslexia (e.g, Goswami et al., 2013), only children with a diagnosis of dyslexia and no history of speech or language impairments were studied. Here, we studied children with a diagnosis of SLI and no history or diagnosis of reading impairments. Nevertheless, as indicated on Table 1, a number of the 45 children with SLI did show impaired reading on our test battery. Table 1 also shows that IQ varied greatly within the SLI group, and that even some participants with very low IQ had preserved reading skills (3 children). Therefore, from this sample of 45 SLI children, we created two sub-groups with intact IQ. Following Fraser et al. (2010), children with SLI were regarded as having non-verbal IQ within the normal range if they scored 80 or above on at least one of the two non-verbal measures (WISC, Ravens). One sub-group comprised a sample of children with pure SLI and no IQ or reading difficulties (*N* = 16), hereafter the “Pure SLI” group. The second sub-group (*N* = 15) comprised a separate sample of SLI children with preserved IQ but reading difficulties, defined as having a SS < 85 on at least two of the standardized reading and spelling tests used. These children also showed phonological difficulties on the experimental measures of phonological processing used (described below), hence hereafter they are termed the “SLI PPR” (poor phonology and reading) group. Note that the SLI PPR children would not qualify for a diagnosis of developmental dyslexia because of their spoken language impairments. As there is no theoretical reason to expect auditory processing ability to vary with I.Q. (see Kuppen et al., 2011), we analyse data for the entire sample of SLI children as well as for these two sub-groups (Pure SLI, SLI PPR).

Fifty CA-matched control children from the same schools as the SLI children also participated in the study. These comprised children who returned consent forms and who were close to individual SLI participants in age. The control group included 21 males and 29 females, with a mean age of 9 years, 4 months. By selecting control children with non-verbal IQ and reading in the normal range, we created a matched sample of typically-developing children for the Pure SLI group (*N* = 16) and for the SLI PPR group (*N* = 15). Group matching for the standardized ability tasks is shown in Table 2 for these two SLI sub-groupings. Table 2 also includes performance on the experimental tests of phonology that were used (see below).

**Table 2:** **Participant characteristics by matched sub-group**.

	**Pure SLI N = 16**	**Controls N = 16**	***F*_(1, 31)_**	**SLI PPR N = 15**	**Controls N = 15**	***F*_(1, 29)_**
Age in months	109.4 (20.8)	106.6 (17.1)	0.2	115.5 (14.0)	107.6 (17.2)	1.9
CELF REC SS^a^^,^^b^	12.9 (4.2)	21.3 (4.0)	32.9^***^	11.7 (4.3)	21.2 (4.2)	37.7^***^
CELF EXPR SS^c^	11.4 (2.8)	18.3 (3.4)	39.2^***^	7.2 (1.6)	18.3 (3.5)	124.0^***^
WISC NVIQ SS^d^	91.1 (19.6)	96.1 (14.5)	0.7	87.8 (14.1)	95.7 (14.9)	2.2
Ravens	95.3 (14.1)	93.8 (10.2)	0.1	83.3 (14.7)	92.3 (8.8)	4.1
BPVS SS^e^	94.2 (9.3)	104.5 (8.6)	10.5^**^	86.6 (10.3)	104.2 (8.9)	25.4^***^
BAS reading SS^f^	101.8 (12.3)	104.8 (10.5)	0.6	73.9 (9.7)	104.5 (10.8)	67.2^***^
TOWRE SS^g^	97.3 (17.0)	102.1 (9.4)	1.0	69.5 (11.0)	101.1 (8.7)	75.9^***^
BAS spelling SS	101.2 (17.6)	106.3 (12.7)	0.9	68.4 (8.9)	104.3 (8.9)	87.6^***^
Oddity rhyme (out of 20)	13.4 (4.3)	15.0 (3.1)	1.5	8.4 (3.0)	14.9 (3.1)	33.3^***^
PSTM^h^ (Words correct)	36.3 (13.5)	41.8 (8.0)	1.9	30.4 (8.1)	42.4 (7.8)	17.1^***^
RAN^j^ (seconds)	45.7 (24.4)	36.4 (7.0)	2.2	55.4 (18.2)	35.7 (6.3)	15.8^***^

Standard deviations in parentheses. *p < 0.05,

** *p < 0.01*,

*** *p < 0.001*.

a *SS, standard score*.

b *Clinical Evaluation of Language Fundamentals, Receptive*.

c *Clinical Evaluation of Language Fundamentals, Expressive*.

d *WISC non-verbal IQ*.

e *British Picture Vocabulary Scales*.

f *British Ability Scales single word reading*.

g *Test of Word Reading Efficiency combined score*.

h *Phonological short-term memory*.

j *Rapid Automatized Naming combined score*.

### Standardized tests

All tests described here were administered in Year 1 of the study except for the reading and spelling tests. Language abilities were measured through the use of two receptive subtests (Concepts and Directions, and Semantic Relations or Sentence Structure, depending on the child's age) and two expressive subtests (Formulating Sentences, and Sentence Assembly or Word Structure, depending on the child's age) of the CELF-3 (Semel et al., 1995; in some tasks the CELF has different versions for children aged 6–9 years, and for children older than 9 years). For all children, receptive vocabulary was measured through use of the BPVS, and single word reading and spelling was assessed using the BAS and TOWRE tests. All children also completed four subscales of the WISC III: Block Design, Picture Arrangement, Similarities and Vocabulary. These four scales yield an estimate of full-scale IQ (pro-rated, see Sattler, 1982), and the two non-verbal scales (Block Design, Picture Arrangement) were used to gain an estimate of non-verbal IQ following the procedure adopted by Sattler (1982, pp. 166–167). Non-verbal IQ was also assessed using the Ravens. There were no significant non-verbal IQ differences between the matched sub-groups, as shown in Table 2.

### Auditory and linguistic tasks

A set of auditory processing tasks using non-speech stimuli (sine tones) or speech stimuli (the syllable “ba,” described further below) were created or adapted for this project by RC, and were all administered during Year 1 of the study. The stimuli were presented binaurally through headphones at 75 dB SPL. Earphone sensitivity was calculated using a Zwislocki coupler in one ear of a KEMAR manikin (Burkhard and Sachs, 1975). The tasks used a “Dinosaur” threshold estimation interface for children in which attractive dinosaur cartoons make noises (originally created by Dorothy Bishop, Oxford University). An adaptive staircase procedure (Levitt, 1971) designed to move rapidly to the child's auditory threshold using a combined 2-down 1-up and 3-down 1-up procedure was used, with a test run terminating after 8 response reversals or the maximum possible 40 trials. The threshold was calculated using the measures from the last four reversals. This indicated the smallest difference between stimuli at which the participant could still discriminate with a 79.4 per cent accuracy rate. The children were assessed individually in a quiet room within their school or at home. A rigorous practice procedure (5 trials) was applied prior to the presentation of the experimental stimuli. For all the Dinosaur tasks (unless otherwise stated below in the individual task descriptions), an AXB paradigm was used; three sounds were presented consecutively, as if they were the sounds made by three distinctive cartoon dinosaurs on screen (500 ms ISI). The middle stimulus (X) was always the standard stimulus and either the first (A) or the last (B) stimulus was different from the standard. At the start of each task, the child was introduced to three cartoon dinosaurs, and for each trial the child was asked to choose which dinosaur produced the target sound i.e., whether A or B was different from X. Feedback was given online throughout the course of the experiment. All the speech stimuli were based on the monosyllable bɑː, and were resynthesized from a natural bɑː token produced by a female native speaker of Standard Southern British English. She was recorded in a sound-attenuated booth; the equipment used was a Tascam DR-100 handheld recorder with an AKG C1000S cardioid microphone. One bɑː token was selected for manipulation and saved in.wav format. Details of the stimulus manipulations, which were done with the software Praat (Boersma and Weenink, 2010), are given below. All “ba” tasks were run with the Dinosaur program, but the cartoon animals that appeared on screen were sheep (because sheep say “baaa”).

#### Amplitude rise time (ART) tasks

**Figure 2:**
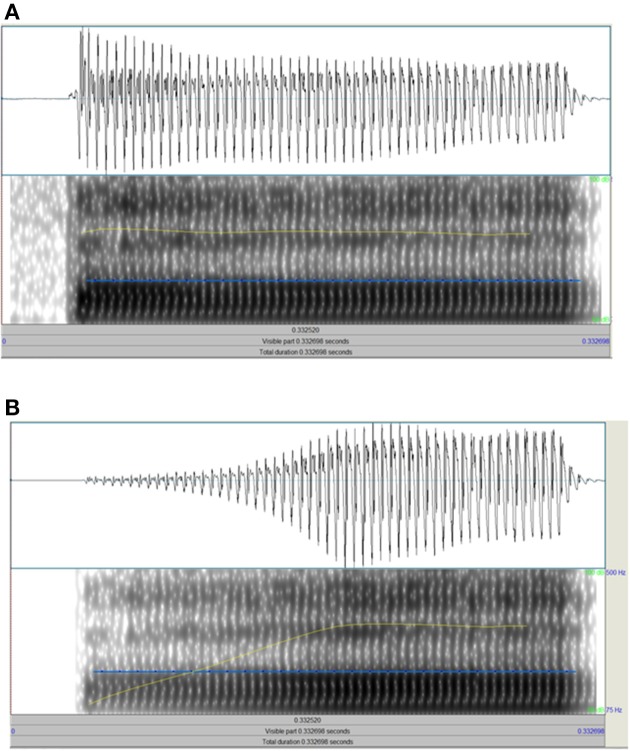
**Schematic depiction of the end points of the “ba” rise time continuum**. **(A)** Shows the standard with 10 ms rise time, **(B)** shows the end point of the continuum with 150 ms rise time. Each panel shows the raw wave form above and the spectrogram below, with the intensity contour in yellow and f0 in blue.

For the non-speech task, three 800 ms sinusoid tones (500 Hz) were presented. The second tone was always a standard tone (X), with a 15 ms linear amplitude rise time, 735 ms steady state, and a 50 ms linear fall time. One of the other two tones was identical to this standard, and the other tone varied in linear amplitude rise time. For this variable amplitude rise time, a continuum of 39 stimuli was used which increased in 7.3 ms steps from the standard to the tone with the longest amplitude rise time at 293 ms. It was explained that each dinosaur would make a sound and that the child's task was to decide which dinosaur made the sound that started off more quietly and got louder more slowly than the other two dinosaurs (longer amplitude rise time). In previous papers by Goswami and colleagues, this task has been called the “1 Rise” task. For the *Speech* task (see Figure 2), three bɑː stimuli with a duration of 300 ms and a flat f0 at 200 Hz were presented. The second bɑː was always a standard stimulus (X), with a 10 ms amplitude rise time (see Figure 2A). One of the other two stimuli was identical to this standard, and the other stimulus varied in amplitude rise time. For this variable amplitude rise time, a continuum of 39 stimuli was used which increased in 3.7 ms steps from the standard to the stimulus with the longest amplitude rise time at 150 ms (see Figure 2B). This continuum was created by copying the original bɑː token 39 times, and resynthesing each copy with a specified amplitude rise time using the *IntensityTier* function in Praat. The standard stimulus also underwent resynthesis from the original token, but without a change of rise time. It was explained that each sheep would make a sound and the child's task was to decide which sheep didn't make a proper “b” sound at the start compared to the other two sheep (longer amplitude rise time). (This instruction was decided on after pilot tests showed it was the best description and children understood what was meant as soon as they heard the practice trials).

#### Duration tasks

For the nonspeech task, three 500 Hz sinusoid tones with a 50 ms linear amplitude rise time and 50 ms linear fall time were presented. The second tone was always a standard tone (X) at 125 ms (note that this is a measure of shorter durations than those used by Corriveau et al., 2007, which varied between 400 and 600 ms). One of the other two tones was identical to this standard, and the other varied in duration. For this variable duration, a continuum of 39 stimuli was used which increased in 3.2 ms steps from the standard to the longest tone at 247 ms. It was explained that each dinosaur would make a sound and that the child's task was to decide which dinosaur made the sound that was longer. For the *Speech* task, three bɑː stimuli with a flat f0 at 200 Hz were presented. The second bɑː was always a standard stimulus (X) at 150 ms. One of the other two stimuli was identical to this standard, and the other stimulus varied in duration. For this variable duration, a continuum of 39 stimuli was used which increased in 3.9 ms steps from the standard to the longest stimulus at 300 ms. This continuum was created by copying the original bɑː token 39 times, and resynthesising each copy with a specified duration using the *DurationTier* function in Praat. The standard stimulus also underwent resynthesis from the original token, but without a change of duration. It was explained that each sheep would make a “baa” sound and that the child's task was to decide which sheep made the “baa” sound that was longer.

#### Frequency (rising f0) tasks

Three 300 ms sinusoid tones with a 5 ms linear amplitude rise time and 5 ms linear fall time were presented. The second tone was always a standard tone (X) with a 10 ms fundamental frequency (f0) rise time from 295 to 500 Hz (hence dynamic f0). One of the other two tones was identical to this standard, and the other tone varied in f0 rise time. For this variable f0 rise time, a continuum of 39 stimuli was used which increased as an exponential function from the standard to the tone with the longest f0 rise time at 150 ms. It was explained that each dinosaur would make a sound and that the child's task was to decide which dinosaur made the sound that started “wobbly” compared to the other two dinosaurs (longer f0 rise time). (This instruction was decided on after pilot tests showed it was the best description and children understood what was meant as soon as they heard the practice trials). For the *Speech* task, three bɑː stimuli with a duration of 300 ms were presented. The second bɑː was always a standard stimulus (X) with a 10 ms f0 rise time from 130 to 220 Hz (hence dynamic f0). (The onset of the f0 rise was the point of vowel onset (as opposed to syllable onset), because f0 would not be perceptible during the silence of the closure and the aperiodicity of the burst releasing the plosive [b].) One of the other two stimuli was identical to this standard, and the other stimulus varied in f0 rise time. For this variable f0 rise time, a continuum of 39 stimuli was used which increased as an exponential function from the standard to the stimulus with the longest f0 rise time at 150 ms. This continuum was created by copying the original bɑː token 39 times, and resynthesiing each copy with a specified f0 rise time using the *PitchTier* function in Praat. The standard stimulus also underwent resynthesis from the original token, but without a change of f0 rise time. It was explained that each sheep would make a sound and that the child's task was to decide which sheep made the sound that started “wobbly” compared to the other two sheep (longer f0 rise time).

#### DeeDee task

This task was developed for children with developmental dyslexia, who show reliable impairments in the task (see Goswami et al., 2010, 2013), and was administered in Year 2 of the study. The DeeDee task used names or titles familiar from children's films and books (e.g., “Harry Potter”), presented using the reiterated syllable “dee.” Four synthesized Dee tokens (stressed [DEE] and unstressed [dee] in initial versus final position) were created that incorporated no cues to phrasal-level constituents. These were then combined into the appropriate sequence for each film or book title used. For example, if the target was “Harry Potter,” the child heard “DEE dee DEE dee.” Accurate performance thus depended on matching an abstract stress pattern to the child's stored rhythmic stress pattern for this target (which should be SWSW). During a pretest, childrens' familiarity with the target stimuli was first ascertained. The children looked at a booklet of pictures that represented the different films and books being used with the experimenter, and named those that they knew. Children were told the names of pictures they did not recognize. The experimental DeeDee task comprising 20 trials was then delivered by computer, with the child listening through headphones in a two alternative forced choice paradigm. The child saw the picture representing the target phrase (e.g., a picture of Harry Potter), and then pressed a button to listen to two DeeDee phrases. One matched the target picture, and the child's task was to choose the DeeDee sequence that they thought matched the picture. Further details are provided in Goswami et al. (2010). Performance in the experimental task was scored as the percentage of pictures recognized in the pretest for which the correct DeeDee sequence was then chosen in the experimental task.

#### Syllable stress perception task

This task was previously used with dyslexic children by Goswami et al. (2013) and was adapted from a task originally created by Leong et al. (2011). It was also administered in Year 2 of the study. Participants listened to a 4-syllable word pronounced twice, and made a same-different judgment about its stress pattern. The words were either given the correct stress pattern on both occasions, or were spoken with a correct and an incorrect stress pattern. For example, for the word pair *DIfficulty* (SWWW) *–diFFIculty* (WSWW), a “different” judgment was required. The task was based on 10 4-syllable words with stress templates that had first syllable stress (such as *caterpillar* and *difficulty*) and 10 4-syllable words with stress templates that had second syllable stress (such as *maternity* and *ridiculous*). The words were selected on the basis of syllable structure (no consonant clusters in the first two syllables), spoken and written frequency and overall familiarity, and did not have alternative pronunciations. The two sets of lexical templates (SWWW, WSWW) were matched as closely as possible for spoken and written frequencies. All items were produced naturally by a native female speaker of British English and recorded for computerized presentation using Audacity and Praat software. Two spoken tokens were recorded for each word. In one token, the speaker emphasized only the first syllable of the word (producing a SWWW stress pattern). In the other token, the speaker emphasized only the second syllable of the word (producing a WSWW stress pattern). Word pairs were then created for each trial by combining the two spoken tokens in all four possible ways, resulting in 80 trials overall. Further details of the task including the acoustic parameters of the stimuli are available in Leong et al. (2011).

### Phonological tasks

Children with SLI who also present with poor reading would be expected to have phonological processing difficulties, whereas the Pure SLI group identified here may not show phonological difficulties. Three experimental measures of phonological processing, previously used with children with dyslexia, were therefore also administered in Year 1 of the study. As shown in Table 2, the Pure SLI group did not show phonological processing difficulties in these tasks compared to control children, whereas the SLI PPR group did show phonological difficulties.

#### Rhyme oddity task

Children listened to sets of three words and had to select the nonrhyme (e.g., boot, *cool*, root; Goswami et al., 2013). The words were presented by computer through headphones using digitized recordings of speech produced by a female native speaker of Standard Southern British English, and trials were presented in one of three fixed random orders. The task comprised 20 trials. Two practice trials with feedback were given prior to the experimental trials.

#### Phonological short-term memory (PSTM) task

The children heard four monosyllabic consonant-vowel-consonant words presented by computer through headphones using digitized recordings of speech produced by a female native speaker of Standard Southern British English (e.g., *type, rib, nook, bud*; originally used in Thomson et al., 2005). The children were required to repeat back the words as spoken. Sixteen trials were presented in total, eight comprising items drawn from dense phonological neighborhoods, and eight trials comprising items drawn from sparse phonological neighborhoods. The total number of items reported correctly out of 64 was used in the analyses.

#### Rapid automatized naming (RAN) task

In the RAN task, children were asked to name line drawings of two sets of familiar objects (first set: *cat, shell, knob, zip, thumb*; second set: *web, fish, book, dog, cup*; see Richardson et al., 2004). For each set, children were first introduced to the names of the pictures and then shown a page with the same pictures repeated 40 times in random order. The children were asked to produce the names as quickly as possible. Average naming speed across the two lists in seconds was used in the analyses.

## Results

Data for the different tasks were explored by group using box plots and the Shapiro–Wilk test to check whether the assumptions of normality were met. Data points lying farther than three interquartile ranges from the nearer edge of the box were removed. After removing the scores of two CA outliers on the syllable stress task and of three SLI children with a *d*' score of 0, and removing the score of one SLI child who scored 0 on the DeeDee task, the prosodic measures met assumptions of normality (kurtosis and skew < 1.96). The auditory threshold distributions remained non-normal even after outliers were removed (all outlier scores from TD controls; three outliers for Ba Duration, three outliers for Ba Rise, and one outlier for Tone F0), hence the bootstrap function in SPSS was used for the auditory processing comparisons and for the multiple regression analyses (1000 permutations, confidence intervals 95%, bias corrected and accelerated). Bootstrapping estimates the properties of the sampling distribution from the sample data, and provides 95% confidence intervals for the mean or other measures of interest. Performance in the DeeDee task and the syllable stress task, and mean auditory thresholds in ms by group, are shown in Table 3. The mean auditory thresholds represent the average difference between the standard stimulus and the varying stimulus that could be detected by each group.

**Table 3:** **Performance in the auditory processing and syllable stress measures by group**.

	**All SLI N = 45**	**All controls N = 50**	**Pure SLI N = 16**	**Pure controls N = 16**	**SLI PPR N = 15**	**PPR controls N = 15**
DeeDee % correct	60.0 (14.0)	70.8 (18.0)	62.7 (15.6)	75.4 (13.1)	63.5 (14.7)	74.2 (12.6)
Syllable stress d'	3.0 (1.2)	4.2 (0.6)	3.6 (1.1)	4.2 (0.7)	3.0 (0.9)	4.2 (0.7)
ART /ba/ ms	46.8 (45.1)	12.1 (3.5)	30.5 (29.4)	13.0 (3.2)	31.3 (27.1)	13.0 (3.4)
ART tone ms	170.3 (83.2)	107.8 (79.7)	161.5 (88.2)	107.2 (85.9)	152.0 (93.2)	108.2 (88.8)
Duration /ba/ ms	63.8 (38.1)	38.6 (20.1)	43.5 (29.1)	33.7 (17.2)	65.2 (35.4)	34.4 (17.6)
Duration tone ms	59.4 (35.0)	37.8 (24.9)	49.5 (33.9)	39.5 (28.5)	42.7 (25.8)	40.8 (29.0)
F0 /ba/ ms	61.4 (40.2)	45.3 (40.3)	45.6 (37.4)	41.1 (35.9)	56.8 (40.2)	43.1 (36.2)
F0 tone ms	40.7 (37.3)	9.6 (6.4)	25.7 (33.9)	10.4 (7.6)	35.2 (33.6)	10.7 (7.7)

*Standard deviations in parentheses*.

### DeeDee task

Accuracy was above chance for all groups and sub-groupings (Pure SLI, SLI PPR). For the whole sample (*N* = 95), an independent samples *t*-test (one-tailed) showed a significant group difference in DeeDee performance, *t*_(1, 93)_ = 3.1, *p* < 0.01; the children with SLI were less accurate than the TD controls. For the Pure SLI group and their TD controls, and for the SLI PPR children and their TD controls, similar *t*-tests also showed that the children with SLI were significantly poorer than the control children, Pure SLI *t*_(1, 30)_ = 2.5, *p* < 0.01; SLI PPR *t*_(1, 27)_ = 2.1, *p* < 0.05. The Pure SLI and SLI PPR groups did not differ, *t*_(1, 28)_ = 0.2.

### Syllable stress perception task

One-tailed independent samples *t*-tests were again used to compare the groups. For the whole sample, the *t*-test showed a significant group difference in sensitivity to syllable stress, *t*_(1, 88)_ = 6.4, *p* < 0.001). For the Pure SLI group and their TD controls, and for the SLI PPR children and their TD controls, similar *t*-tests also showed that the children with SLI were significantly poorer than the control children, Pure SLI, *t*_(1, 29)_ = 1.8, *p* < 0.05; SLI PPR *t*_(1, 27)_ = 4.1, *p* < 0.01. The difference between the Pure SLI and SLI PPR groups approached significance, *t*_(1, 29)_ = 1.7, *p* = 0.051; the SLI PPR children tending to perform more poorly in the lexical stress task.

### Basic auditory processing

For the whole sample (*N* = 95), group comparisons using bootstrapped independent samples *t*-tests (two-tailed) showed significant group differences for each auditory variable, with the children with SLI showing higher thresholds in each case (all *p*'s < 0.001, except for Ba f0 comparison where *p* < 0.01). When comparing the Pure SLI children and their TD controls, a bootstrapped independent samples *t*-test for each measure showed a significant group difference for hearing rise time in speech only [Ba rise, *t*_(1, 27)_ = 2.7, bootstrapped *p* < 0.05]. The group difference in hearing rise time in non-speech tones approached significance [*t*_(1, 30)_ = 1.8, bootstrapped *p* = 0.088]. For the other auditory measures, group performance was statistically equivalent for the Pure SLI and the TD children. For the SLI PPR comparisons, a similar bootstrapped independent samples *t*-test for each measure showed significant group differences for hearing duration and rise time in speech [Ba duration, *t*_(1, 28)_ = 3.0; Ba rise, *t*_(1, 25)_ = 2.6], and for non-speech dynamic f0 [*t*_(1, 28)_ = 3.0, all bootstrapped *p*'s <0.05]. Discrimination of rise time for non-speech stimuli did not reach significance for this sub-group comparison, an unexpected result given the prior literature [*t*_(1, 28)_ = 1.4]. Nevertheless, inspection of Table 3 shows that auditory perception was always poorer (higher thresholds) for the SLI children. Inspection of Table 3 also suggests that whereas both SLI groups with preserved NVIQ showed difficulties in hearing ART, the Pure SLI group was better than the SLI PPR group regarding auditory sensitivity to duration and rising f0 in speech (Ba duration, Ba f0). A set of bootstrapped independent samples *t*-tests (one tailed) hence compared auditory thresholds for the Pure and PPR groups of children with SLI and intact NVIQ. Significantly greater impairments in perceiving duration in speech were found for the PPR group, *t*_(1, 29)_ = 1.8, *p* < 0.05. The apparent group difference in sensitivity to rising f0 in speech (see thresholds in Table 3) did not reach statistical significance, *t*_(1, 29)_ = 0.6.

As the different measures of auditory sensitivity to rise time, duration and rising f0 were administered in Year 1 of the study while the prosodic measures were administered in Year 2, partial time-lagged rank-order correlations were used (Spearman's rho) to see whether auditory processing was predictive of success in the prosodic tasks administered a year later. Children's prosodic sensitivity indeed showed significant time-lagged associations with their earlier auditory sensitivity (see Table 4). As would be expected, performance in the DeeDee task was significantly related to performance in the syllable stress perception task, *r* = 0.38, *p* < 0.001. In order to see whether the auditory measures made differing contributions to performance in the two measures of prosodic sensitivity, multiple regression analyses were used. Only the auditory speech measures (the Ba tasks) were used as predictors, as all children were more sensitive to the auditory parameters of ART, duration and rising f0 in the speech measures. Non-verbal IQ and age were also included in the equations as predictors. One equation used DeeDee performance as the dependent measure, and the second equation used syllable stress *d*'. All five predictors (Ba ART, Ba duration, Ba f0, NVIQ, age) were entered together, and the Beta values with standard errors and confidence intervals, the standardized Beta values and the bootstrapped *p*-values are shown in Table 5.

**Table 4:** **Time-lagged partial correlations between performance on the syllable stress tasks given in Year 2 and the auditory processing measures given in Year 1, controlling for NVIQ and age**.

	**Ba duration**	**Ba F0**	**Ba ART**	**Tone duration**	**Tone F0**	**Tone ART**
DeeDee	−0.39^***^	−0.37^***^	−0.32^**^	−0.36^***^	−0.34^***^	−0.25^*^
Syllable stress	−0.50^***^	−0.37^***^	−0.52^***^	−0.44^***^	−0.45^***^	−0.40^***^

*** *p < 0.001*,

** *p < 0.01*,

* *p < 0.05*.

**Table 5:** **Multiple regression equations with performance on the syllable stress and DeeDee tasks as the dependent variables and auditory processing, NVIQ, and age as predictors**.

	**DeeDee Ƅ (CI)**	**Ƅ SE**	**ß**	**Sig**	**Syll stress Ƅ (CI)**	**Ƅ SE**	**ß**	**Sig**
NVIQ	0.021 (−0.16 to −0.20)	0.09	0.03	0.84	0.01 (0 to 0.03)	0.01	0.27^**^	0.002
Age	0.15 (−0.05 to 0.30)	0.10	0.14	0.16	0.01 (−0.01 to 0.01)	0.01	0.04	0.63
Ba ART	−0.29 (−0.65 to −0.08)	0.17	−0.16	0.19	−0.04 (−0.07 to −0.02)	0.01	−0.37^**^	0.000
Ba f0	−0.47 (−0.86 to −0.11)	0.19	−0.27^*^	0.018	−0.01 (−0.02 to 0.01)	0.01	−0.07	0.35
Ba dur	−0.31 (−0.82 to 0.16)	0.25	−0.14	0.27	−0.04 (−0.07 to −0.01)	0.01	−0.30^**^	0.001

*Ƅ (CI), unstandardized beta and confidence intervals; Ƅ SE, standardized error for Ƅ; ß, standardized beta coefficient; Sig, bootstrapped significance; Syll stress, syllable stress*.

* *p < 0.05*;

** *p < 0.01*.

The multiple regression analysis for the DeeDee task accounted for a significant 48% of variance in the task, and showed only one significant predictor, hearing dynamic frequency changes in speech (Ba f0). In contrast, the multiple regression analysis for the syllable stress task accounted for a significant 77% of variance in the task, and revealed three significant predictors, Ba ART, Ba duration and non-verbal IQ. The regression analyses suggest that different auditory cues contribute to individual differences in processing lexical stress (the syllable stress misperception task) and abstract stress patterns (the DeeDee task). While sensitivity to dynamic pitch change (rising f0) seems most important for perceiving abstract stress patterns as measured by the DeeDee task, sensitivity to ART and duration were most important for perceiving lexical stress, at least for the tasks used here. A final pair of regression equations looked at the significant predictors of performance on the clinical language measure employed (the CELF), with one equation predicting performance on the Receptive language measures and one equation predicting performance on the Expressive language measures (SLI children only). The independent variables in each equation were entered in three blocks, and were (1) age and non-verbal IQ, (2) Ba ART, Ba f0 and Ba duration, and (3) accuracy in the DeeDee task and syllable stress *d*'. Both equations showed only two significant predictors, NVIQ and syllable stress *d*'. The equation for receptive language skills accounted for 74% of the total variance, with a standardized Beta value of 0.55 for NVIQ and 0.51 for syllable stress *d*', respectively, both *p's* < 0.01 (bootstrapped *p*-values). The equation for expressive language skills accounted for 77% of the total variance, with a standardized Beta value of 0.50 for NVIQ and 0.48 for syllable stress *d*' respectively, both *p's* < 0.01 (bootstrapped *p*−values).

## Discussion

Here we set out to investigate a perceptual hypothesis regarding the etiology of developmental SLIs based on impaired sensitivity to amplitude envelope rise times and associated prosodic sensitivity. A similar perceptual hypothesis proposed in the 1990s (the “surface” hypothesis, McGregor and Leonard, 1994) suggested that children with SLIs had difficulty in learning grammatical morphology because they had prosodic difficulties arising from auditory insensitivity to morphemes that were short in duration and of low intensity. The new form of this perceptual hypothesis proposed here, the “prosodic phrasing” hypothesis, is that auditory difficulties in the accurate processing of ARTs cause impairments in detecting the global prosodic or rhythmic phrasing of specific utterances, which leads to language learning difficulties that manifest primarily as problems in processing morpho-syntax. The impaired sensitivity to ARTs in children with SLIs is also hypothesized to affect efficient neural entrainment to the speech signal, perhaps via impaired phase alignment at the “stressed syllable” (~2 Hz) and “syllable” (~5 Hz) rates, with consequent effects on oral language processing. Here we tested the behavioral elements of the prosodic phrasing hypothesis by studying the relationships between sensitivity to ART and prosodic sensitivity in English-speaking children with SLIs.

In the relatively large sample of children with SLIs studied here (*N* = 45), we found significant impairments in both of our behavioral measures of prosodic sensitivity, the DeeDee task (a measure of sensitivity to abstract stress patterns) and the stress misperception task (a measure of sensitivity to lexical stress). These prosodic difficulties were found whether the children with SLI were characterized as having a “pure” form of SLI (with intact IQ and phonology), or whether they had impaired language and phonology (SLI PPR), or whether they had impaired IQ and impaired language. Furthermore, while children with Pure SLI were only significantly impaired in one of the auditory processing measures, that testing sensitivity to ART in speech (the syllable “ba”), children with intact IQ but impaired language and phonology (SLI PPR) were impaired at hearing both ART and duration in speech. Indeed, the PPR group impairment in discriminating duration in speech was significantly greater than that of the Pure SLI children. This is consistent with prior studies of auditory sensitivity in children with SLIs, in which significant impairments in sensitivity to both ART and duration have been reported (Corriveau et al., 2007; Richards and Goswami, 2015). It also suggests that impairments in perceiving *duration* as well as ART are important for the accurate detection of prosodic phrasing, requiring the prosodic phrasing hypothesis to be amended to include both ART and duration. When *both* duration discrimination and ART discrimination are impaired, as was the case for the majority of children in our SLI sample, then children with SLI seem to exhibit *both* grammatical and phonological deficits (see also Corriveau et al., 2007). The importance of duration is also consistent with findings in adult studies of auditory factors in prosodic prominence, where although the phrasal metrical pattern (here thought to be governed by sensitivity to ART) is more important than syllable duration *per se* for detecting perturbations in speech rhythm, both factors play a role in successful performance (Zheng and Pierrehumbert, 2010). Meanwhile, children with SLI and low IQ were impaired in all of the auditory tasks used. This finding suggests a relationship between the severity of auditory impairments and the severity of language impairments, at least in the current sample.

In order to investigate the nature of the relationships between auditory processing, prosodic sensitivity and language skills, a series of multiple regression equations were constructed and tested using a bootstrapped model (1000 permutations, confidence intervals 95%, bias corrected and accelerated). The regression analyses showed that the only significant auditory predictor of sensitivity to abstract stress patterns (the DeeDee task) was sensitivity to rising f0 in speech. For the lexical stress task (the syllable stress misperception task), the significant auditory predictors were sensitivity to ART and to duration in speech. Hence in this study, different auditory predictors were related to the two types of prosodic sensitivity being measured, both of which contribute to sensitivity to prosodic phrasing. Rising f0 has not been measured in prior studies using the DeeDee task, and deserves further investigation. In the current study, children with SLIs and preserved NVIQ were not impaired in hearing rising f0 compared to TD children. As children with low NVIQ are not typically included in studies of developmental SLIs, the relationship between rising f0 and DeeDee performance may be specific to the current sample.

In a second set of regression analyses, the relationship between the behavioral measures and SLIs was investigated by entering the sensory variables (performance in the auditory tasks) as a block after age and NVIQ, and then the prosodic variables as a further block. In these equations, sensitivity to lexical stress (the syllable stress *d*' measure) and NVIQ were the only significant predictors of language skills (the childrens' CELF standard scores), for both expressive and receptive language. These data suggest that the DeeDee task may be of limited value as a measure of children's sensitivity to prosodic information in natural language (note, however, that it is a useful task for *training* prosodic sensitivity, see Bhide et al., 2013; Thomson et al., 2013). The data also suggest that a fresh look at the role of perceptual sensitivity to lexical stress might be a useful research strategy for explaining the range of morpho-syntactic deficits that characterize SLIs across languages.

It is important to emphasize, however, that impaired sensitivity to ART was not the only auditory deficit found in the sample of children with SLI studied here. Although ART was the only auditory deficit to reach significance when both NVIQ and phonological processing were intact (the Pure SLI group), when NVIQ was preserved but phonological processing was impaired then sensitivity to duration cues in speech and to rising f0 in tones was also significantly impaired. Further, inspection of Table 3 shows that the children with SLI were less sensitive to all the auditory measures used whatever grouping was employed, and indeed all of the auditory tasks showed significant time-lagged correlations with the prosodic measures (Table 4). Therefore, for this sample of children, auditory processing in general was impaired in comparison to age-matched controls. Clearly, there are strong associations between poor auditory processing, poor awareness of lexical stress, and poor spoken language development. Investigation of whether these phonological difficulties with syllable stress can be linked *systematically* to the morpho-syntactic errors made by children with SLIs across languages awaits further study.

Note also that this form of the perceptual hypothesis links phonological processing difficulties at the supra-segmental level directly to the grammatical deficits observed in children with SLIs. The prosodic phrasing hypothesis does not propose a direct link between linguistic processing and grammatical knowledge that is independent of sensory information and governed by a “gene for grammar” (e.g., van der Lely and Pinker, 2014). Rather, the perceptual deficits identified are thought to impair the development of morpho-syntactic knowledge because grammatical knowledge is learned in part on the basis of phonological (prosodic) structure. Indeed, it is interesting that the original developmental study of inflectional morphology in children, conducted by Berko (1958), was also suggestive of a key role for phonology. Berko used a pictorial nonword elicitation task to encourage typically-developing children of high ability to produce inflectional morphemes (e.g., *wug–wugs* [plural]; *quirky–quirkier–quirkiest;* [adjectives]). While the preschool children that she studied were very successful with some phonological forms (e.g., 91% were successful with *wug–wugs*), the children applied the same inflectional rule quite poorly to other phonological forms (only 28% were successful with *nizz–nizzes*, and 36% with *gutch–gutches*). These enormous differences in success for typically-developing pre-schoolers (the children were at Harvard University preschool) are consistent with a phonological influence on successful performance. Phonological neighborhood analyses of spoken English show that while there are many phonological rhyme neighbors for an item like *wug* (*bug, rug, mug, jug*.., 19 rhyme neighbors, see De Cara and Goswami, 2002), there are fewer phonological neighbors for *nizz* (only eight, such as *his*, *fizz* and *whizz*, which are not nouns) and also fewer phonological neighbors for *gutch* (only seven, such as *much, touch*, and *hutch*; De Cara and Goswami, 2002). If morphology and phonology are associated in typical development, then they are also likely to be associated in atypical development. It would be interesting to repeat Berko's study and also measure prosodic sensitivity and basic auditory processing in participating TD children, in order to explore the nature of these relationships in more detail.

Note further that although we find impairments in perceiving ART and in perceiving syllable stress in both dyslexic children (e.g., Goswami et al., 2010, 2013) and in children with SLIs (Richards and Goswami, 2015, and the current study), the etiology of the two developmental disorders may still be distinct. While some studies report a high degree of overlap in the children receiving diagnoses of SLI and developmental dyslexia (around 50%, see McArthur et al., 2000), and while the severity of the perceptual difficulties with ART is related to language processing in some studies comparing the two disorders (see Fraser et al., 2010; Johnson et al., 2011), there are likely to be multiple developmental factors at play. Indeed, our view is that a developmental and multi-modal perspective is required to study causality in developmental language disorders. For example, both dyslexia and SLI involve aspects of oral language processing, with a primary deficit in phonology characterizing children with developmental dyslexia, and a primary deficit in morpho-syntax characterizing children with SLI. If the neural multi-time resolution models of speech processing (e.g., Giraud and Poeppel, 2012) are accurate and human speech encoding depends on oscillatory entrainment to different rates of AM in speech, then impairments in perceiving ARTs (particularly at slower AM rates below 10 Hz, see Goswami, 2015) would be expected to be associated with impairments in oral language processing and to cause impairments in prosodic sensitivity in both developmental disorders. However, acoustic rhythm is also encoded by motor cortex in the human brain (e.g., Grahn and Brett, 2007), and this is probably also true (although not yet tested experimentally) for infants and children (e.g., Tierney and Kraus, 2014). Further, whereas children with developmental dyslexia often have age-typical processing of sound duration, children with SLIs usually do not (e.g., Corriveau et al., 2007; Goswami et al., 2010; Huss et al., 2011; Richards and Goswami, 2015).

There is also *visual* encoding of speech rhythm, as shown for example in the research on sensitivity to “visual prosody” in adults (e.g., Munhall et al., 2004). Adult studies have shown that the rhythms of speech are visible on the face, which accurately mirrors changes in the vocal tract as speech is produced (Ghazanfar and Takahashi, 2014a). Facial deformations while speaking (low-frequency temporal movements of the cheeks and face) are tightly correlated with acoustic output, and both mouth motion and the speech AE exhibit a 3–8 Hz rhythm, related to the rate of syllable production by adults (Chandrasekaran et al., 2009). This “visual prosody” is thought to support the perception and parsing of long-duration vocal signals (Ghazanfar and Takahashi, 2014b), making it potentially crucial for morphological development. Therefore, an acoustic insensitivity to ART alone or accompanied by an insensitivity to duration (as found here) may have different effects developmentally to an acoustic insensitivity to ART accompanied by a motor impairment involving rhythm, or accompanied by a visual impairment involving visual prosody. To date, children with developmental dyslexia appear to have difficulty with motor rhythms and musical rhythms as well as with speech rhythm (see Goswami, 2015, for a recent overview). Yet comparable research with children with SLIs is missing. Sensitivity to visual prosody has yet to be studied in either developmental disorder (although see Megnin-Viggars and Goswami, 2013, for adult dyslexics). Similarly, while neural entrainment to rhythmic speech has been shown to be impaired in children with dyslexia (Power et al., 2013), neural entrainment studies involving children with SLI have yet to be carried out. Given the multi-modal experiences with language known to be critical to language acquisition by infants (e.g., Kuhl, 2004), the study of developmental SLIs is wide open for a multi-modal and neural approach to the development of rhythmic entrainment and possible causal links to developmental language disorders.

In conclusion, the prosodic phrasing hypothesis proposed here offers a new perceptual approach to understanding the etiology of developmental SLIs, across languages. The prosodic phrasing hypothesis argues that auditory insensitivity to ART cues in speech (especially if accompanied by insensitivity to duration cues) causes children difficulties with accurate prosodic representation. Following adult theorists (Frazier et al., 2006), the global prosodic representation is considered to be the skeletal structure upon which understanding utterances depends (see also Goswami and Leong, 2013). Accordingly, children who have inefficient sensory processing of the temporal positions and relative perceptual prominence of the syllable “beats” in speech have associated difficulties with the efficient representation of prosodic phrasing, leading to language difficulties. Over development, TD children seem likely to use prosodic phrasing to develop implicit knowledge of the higher-order consistencies that comprise grammar (metrical expectancy), so that later in development they can predict where stress should fall on the basis of syntactic structure alone (and as adults, they can extract syntactic information even when words are manipulated so that all words are stressed to the same extent, e.g., Pitt and Samuel, 1990). Supporting the perception of speech rhythm in children with SLIs by using music may facilitate the perception of prosodic phrasing (e.g., Jentschke et al., 2008; Jentschke and Koelsch, 2009), however this is currently an open question. In children with reading difficulties, phonological interventions based on music and rhythm have shown promising effects, with increased efficiency of beat entrainment (tapping to a rhythm) linked to individual gains in reading (Bhide et al., 2013). The perceptual difficulties with prosodic phrasing found in children with SLIs may also be improved by rhythm-based interventions that support accurate entrainment. Finally, the perceptual difficulties with prosodic phrasing found here in children with SLIs should also be related to impaired neural entrainment to the rhythm patterns in speech, however this neural part of the prosodic phrasing hypothesis remains to be tested.

### Conflict of interest statement

The authors declare that the research was conducted in the absence of any commercial or financial relationships that could be construed as a potential conflict of interest.
